# Preparation and Electromagnetic Wave Absorption Properties of N-Doped SiC Nanowires

**DOI:** 10.3390/ma16175765

**Published:** 2023-08-23

**Authors:** Ranran Shi, Zheng Liu, Wenxiu Liu, Jianlei Kuang

**Affiliations:** 1 Department of Inorganic Nonmetallic Materials, School of Materials Science and Engineering, University of Science and Technology Beijing, Beijing 100083, China; 2 Beijing Institute of Aeronautical Materials, Aero Engine Corporation of China, Beijing 100095, China

**Keywords:** SiC nanowires, element doping, composite coating, dielectric properties, electromagnetic wave absorption

## Abstract

Enhancing the conductivity loss of SiC nanowires through doping is beneficial for improving their electromagnetic wave absorption performance. In this work, N-doped SiC nanowires were synthesized using three different methods. The results indicate that a large amount of Si_2_ON will be generated during the microwave synthesis of SiC nanowires in a nitrogen atmosphere. In addition, the secondary heat-treatment of the as-synthesized SiC nanowires under nitrogen atmosphere will significantly reduce their stacking fault density. When ammonium chloride is introduced as a doped nitrogen source in the reaction raw material, the N-doped SiC nanowires with high-density stacking faults can be synthesized by microwave heating. Therefore, the polarization loss induced by faults and the conductivity loss caused by doping will synergistically enhance the dielectric and EMW absorption properties of SiC nanowires in the range of 2–18 GHz. When the filling ratio of N-doped SiC nanowires is 20 wt.%, the composite shows a minimum reflection loss of –22.2 dB@17.92 GHz, and an effective absorption (RL ≤ –10 dB) bandwidth of 4.24 GHz at the absorber layer thickness of 2.2 mm. Further, the N-doped SiC nanowires also exhibit enhanced high-temperature EMW absorption properties with increasing temperature.

## 1. Introduction

SiC has excellent mechanical properties, chemical inertness, corrosion resistance and high-temperature oxidation resistance. It also exhibits attractive properties in the fields of optics, electricity and catalysis [1,2,3,4]. Therefore, it is widely used in electronic devices, semiconductors, catalysts, nuclear energy, space and other important fields. Most attractively, its dielectric loss and electromagnetic wave (EMW) absorption performance also increase with increasing temperature [5]. Therefore, SiC is considered as a potential high-temperature EMW absorbing material. However, SiC has no magnetic loss capability and its conductivity loss capability is weak, which limits its EMW absorption performance. According to Debye’s theory, the dielectric loss *ε*″ consists of polarization loss and conductivity loss, as shown in the following equation [6]:

(1) $$\varepsilon \prime \prime =\varepsilon_{p}^{\prime \prime }+\varepsilon_{c}^{\prime \prime }=(\varepsilon_{s}-\varepsilon_{\infty })\frac{\omega \tau }{1+\omega^{2}\tau^{2}}+\frac{\sigma \left(T\right)}{2\pi f\varepsilon_{0}}$$

where *ε_p_* is the polarization loss, *ε_c_* is the conductivity loss, *ε_s_* is the static permittivity, *ε_∞_* is the relative permittivity, *ε*_0_ is the dielectric constant in vacuum, *ω* is the angular frequency, *τ* is the relaxation time, *f* is the frequency and *σ*(T) is the temperature-dependent electrical conductivity. It is clear that increasing the electrical conductivity of SiC nanowires will enhance its conductivity loss. Elemental doping has been shown to be a method used to effectively improve the conductivity loss of SiC by transforming SiC into *n*/*p*-type semiconducting materials, which can also simultaneously enhance the polarization loss by inducing lattice defects [7,8,9,10,11,12]. Among many doped elements, nitrogen doping is a typical method to prepare n-type SiC [13,14].

There are two main methods for improving the dielectric and EMW absorption properties of SiC by nitrogen doping. The first method is to introduce nitrogen sources, including nitrogen and ammonium chloride (NH_4_Cl), into the SiC synthesis process [15,16]. Dou et al. prepared N-doped SiC nanopowders by combustion synthesis using Si, carbon black and PEFE powder as raw materials in a high-pressure Ar-N mixed atmosphere [17]. Su et al. improved the dielectric properties of SiC powders by combustion synthesis using NH_4_Cl as a dopant [18]. The second method is high-temperature heat-treatment nitriding of SiC in a nitrogen atmosphere. Dong et al. prepared N-doped SiC nanoparticles by annealing in N_2_ atmosphere at 1500 °C with different dwelling times, and achieved a maximum nitrogen doping content of 5.25 at.% [19].

The current research mainly focuses on the enhancement of the EMW absorption properties of equiaxed SiC granular materials by nitrogen doping. The dielectric and EMW absorption properties of N-doped one-dimensional SiC are rarely reported. In fact, one-dimensional SiC has significant advantages in the field of EMW absorption. It can construct three-dimensional conductivity loss networks, and then induce higher conductivity loss capabilities after nitrogen doping. Electromagnetic wave absorbing materials are usually composed of absorbing materials (SiC, carbon, magnetic metal, etc.) and wave-transmitting matrix materials (polymer, Si_3_N_4_, glass, etc.). One-dimensional SiC can also effectively enhance the mechanical properties of wave-transmitting matrix materials to form functional and structural integrated materials.

Microwave heating synthesis is an attractive method of material preparation that is fundamentally different from the traditional heating synthesis method. It acts directly on the heated material itself, which can reduce the energy required for the reaction, accelerate the reaction rate, reduce the reaction temperature, improve the reaction yield, and achieve better physical and chemical properties. This is mainly due to the fact that the diffusion rate of atoms and molecules in the microwave field has been significantly improved, which is also conducive to the rapid diffusion of doped atoms in the SiC lattice [20,21,22].

Therefore, in this work, N-doped SiC nanowires were synthesized by three different doping methods through microwave heating. Their dielectric properties and electromagnetic wave absorption properties were carefully investigated and compared. The optimized N-doped SiC nanowires were then combined with resin to form a composite coating. The high-temperature electromagnetic wave absorption properties of the composites were then investigated. The mechanism of EMW absorption is also discussed.

## 2. Materials and Methods

### 2.1. Preparation of N-Doped SiC Nanowires and Composite Coatings

SiC nanowires were prepared using the silica sol (*w*SiO_2_ = 30%) and activated carbon (AR grade, <50 µm, Beijing-Chem Co., Beijing, China) as silicon and carbon sources, respectively. The raw materials were mixed and stirred for 3 h and then were dehydrated at 250 °C for 24 h; the dried mixture was homogenized by ball-milling for 8 h to obtain the starting powder; then the SiC nanowires were synthesized at 1500 °C (heating rate ≥ 50 °C/min) for 30 min by using a 2.45 GHz microwave furnace in a flowing argon (>99.995%) atmosphere; after that, nanowires were concentrated through a gravity concentration process [23].

In this work, the preparation of three different types of N-doped SiC nanowires was achieved by modifying some of the synthesis processes, as shown in Table 1. The SN-A sample was synthesized using a nitrogen (>99.995%) atmosphere instead of an argon atmosphere during the microwave synthesis process. The SN-B sample was prepared by nitriding as-synthesized SiC nanowires under a nitrogen atmosphere at 1200 °C for 30 min. Finally, ammonium chloride (NH_4_Cl, AR grade, ≥99.5%, Beijing-Chem Co., Beijing, China) was introduced as a nitrogen source during the raw material mixing stage to prepare SN-A samples. At high temperature, ammonium chloride will decompose into nitrogen gas, as shown in Equation (2). All samples were decarburized in air at 700 °C for 2 h, and then were acid-washed in HF-HNO_3_ mixtures (HF/HNO_3_=1:1 in molar ratio) to remove SiO_2_.

(2) $$2NH_{4}\mathrm{Cl}=N_{2}+4H_{2}+Cl_{2}$$

In order to investigate the high-temperature EMW absorption performance, N-doped SiC nanowire/resin composite coatings were prepared. DOWSIL™ RSN-0805, which can be used at high temperatures up to nearly 650 °C, was selected as the resin raw material. SiC nanowires and resin were mixed well under high-speed stirring according to the mass ratio of 20:80, and then the air bubbles generated by stirring were eliminated in a vacuum chamber. Subsequently, the composite coating was prepared on the surface of the aluminum plate (100 × 100 mm) using a film applicator and held at 250 °C to achieve complete curing. The 100 × 100 × 2.2 mm SiC nanowire/resin composite coating was finally obtained.

### 2.2. Characterization

The phase compositions and crystal structure were determined by X-ray diffraction with CuKα radiation (XRD, D8 Advance, Bruker AXS, Karlsruhe, Germany). The micro morphologies were observed by field-emission scanning electron microscopy (FESEM, ZEISS Ultra 55, Oberkochen, Germany) equipped with energy dispersive spectroscopy (EDS, Oxford Instruments X-Max, Oxford, UK) and transmission electron microscopy (TEM, JEM 2100F, JEOL, Tokyo, Japan), respectively. The dielectric permittivity in the 2–18 GHz range was measured with an Agilent N5230A vector network analyzer (Agilent, Palo Alto, Canada) using the coaxial method, and performed at room temperature. And the mixtures of SiC and paraffin wax (weight ratio of 2:8) were poured into the measuring ring with the size of 7 × 3 × 2 mm (outside diameter × inside diameter × thickness). The arc method was used to measure the actual EMW absorption properties of the composite coatings at high temperature in the frequency range of 2–18 GHz [24]. The temperature range was from room temperature to 300 °C, and the interval was 100 °C. The coated aluminum plate was placed on an electronic hot plate and subsequently heated up until the coating surface reached a set temperature and held for 5 min. Then, the actual reflection loss curves of the composite coating were collected by the vector network analyzer.

## 3. Results and Discussion

**Figure 1:**
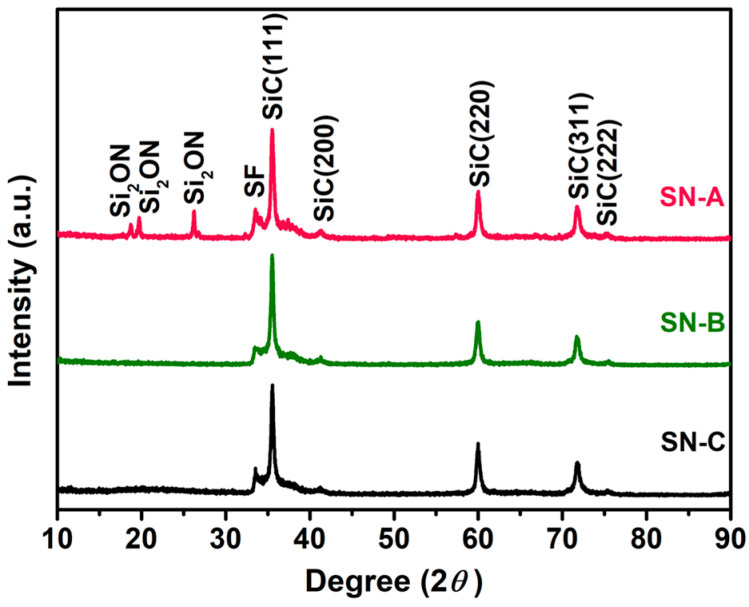
XRD patterns of the N-doped SiC nanowires prepared by different doping methods.

The phase composition of three groups of N-doped SiC nanowires is shown in Figure 1. It can be seen that not only 3C-SiC (JCPDS No. 29-1129) exists in the SN-A sample, but also a large amount of Si_2_ON is formed. This is attributed to the reaction of N_2_ with SiO_2_, the intermediate product SiO and target product SiC to form Si_2_ON during carbothermal reduction [25]. The SN-B sample is entirely composed of 3C-SiC, which is due to the fact that heat treatment at 1200 °C is insufficient to promote the production of nitrogen compounds. The phase composition of the SN-C sample is also monophasic 3C-SiC. Compared with the preparation of SN-A samples in a complete nitrogen atmosphere, the nitrogen generated by the decomposition of ammonium chloride only accounts for a low proportion of the total protective atmosphere, making it difficult to generate Si_2_ON in SN-C samples. Due to the generation of the impurity phase in the SN-A sample, the following research focuses only on SN-B and SN-C samples.

The lattice parameters and stacking fault (SF) density of N-doped SiC nanowires were calculated from the XRD results, as shown in Table 2. The covalent radius of the N atom (0.068 nm) is smaller than that of the C atom (0.077 nm). Therefore, the substitution of N atoms for C atoms in the SiC lattice results in a decrease in the cell volume of SN-B and SN-C samples [26]. The intensity ratio of the SF peak to SiC (200) peak is commonly used to evaluate the density of stacking faults [27]. And high-density stacking faults have been shown to enhance the interface polarization loss. The SFs were formed by embedding 2H-SiC segments in the 3C-SiC matrix with the characteristic of type II heterostructures, which results in the electrons and holes being confined to the opposite sides of the 3C/2H-SiC interfaces, respectively. As a result, a large number of interfacial dipoles are formed, resulting in a strong dipole polarization loss. [28,29]. The calculated SF density shows that compared with pristine SiC nanowires, the SF density of SN-C sample decreases slightly to 2.34, while that of the SN-B sample decreases to a lower value of 2.06. During the preparation of the SN-C sample, a liquid phase of silicon–oxygen–nitrogen will be formed, which is favorable to stabilize the 3C-SiC phase, and may even lead to a reverse transformation from hexagonal SiC to cubic SiC [30,31,32]. This means that the content of 2H-SiC, which constitutes stacking faults, will be reduced. As a result, the SFs density of the SN-C sample is reduced. For the SN-B sample, the secondary heat treatment at 1200 °C for 30 min in the microwave furnace is responsible for the decrease in the SF density [29,33].

**Figure 2:**
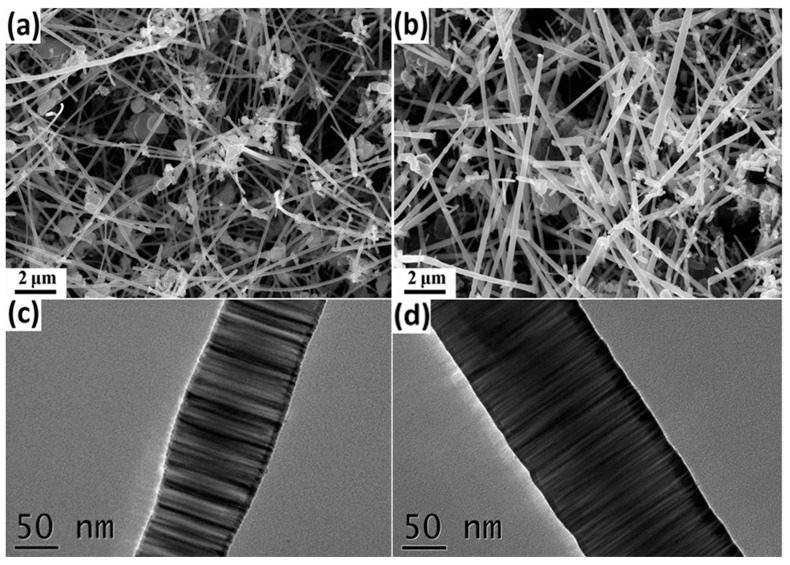
SEM and TEM images of (**a**,**c**) SN-B and (**b**,**d**) SN-C samples.

Figure 2a,b are SEM images of SN-B and SN-C samples, respectively. Due to the absence of a metal catalyst, the SiC nanowires were formed through vapor-solid (VS) mechanisms. Both types of SiC nanowires exhibit straight and smooth morphologies. And their diameter and length are hundreds of nanometers and more than ten microns, respectively. The EDS analysis results indicate that the N element content in SN-B and SN-C samples is 1.24 at.% and 2.55 at.%, respectively. This difference may be attributed to the easier diffusion of N atoms into the lattice of SiC nanowires during their growth relative to the completion of SiC nanowire growth. The SiC nanowires were further observed by transmission electron microscopy, as shown in Figure 2c,d. A large number of stripes perpendicular to the growth direction of SiC nanowires can be clearly observed, indicating that numerous stacking faults are generated in SN-B and SN-C nanowires samples, which is consistent with the calculated SF density.

**Figure 3:**
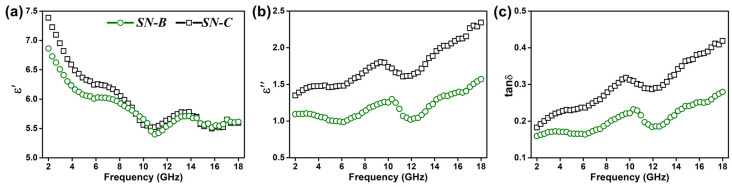
Permittivity properties of N-doped SiC nanowires as a function of frequency: (**a**) dielectric constant, (**b**) dielectric loss, and (**c**) loss angle tangent.

**Figure 4:**
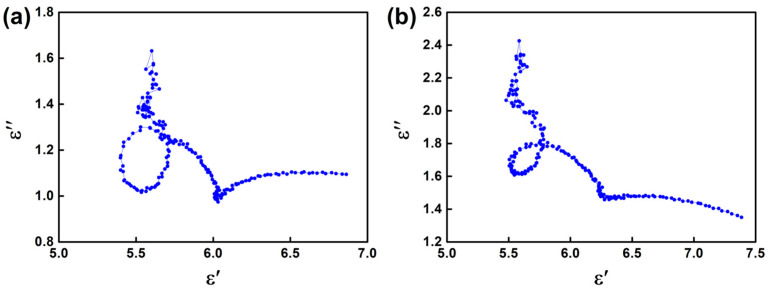
Cole–Cole curves of samples (**a**) SN-B and (**b**) SN-C.

The dielectric parameters of two groups of N-doped SiC nanowire samples were characterized in the frequency range of 2–18 GHz, and the results were plotted in Figure 3. The two different doping methods result in significant differences in dielectric properties. It can be seen that the dielectric constant (*ε*′), dielectric loss (*ε*″) and loss angle tangent (tanδ = *ε*″/*ε*′) of the SN-C samples are higher than those of the SN-B sample, reaching 5.6~7.5, 1.4~2.3 and 0.18~0.42, respectively. There may be two reasons for this. On the one hand, the lower density of SFs results in a lower polarization loss for the SN-B samples. On the other hand, less N elemental doping implies a lower carrier concentration, which also leads to a lower conductivity loss in the SN-B samples. However, the dielectric constant and dielectric loss of both groups of samples are higher than those of the pristine SiC nanowires (*ε*′: 4.9~6.2, *ε*″: 0.9~1.1) [34]. Based on *ε*′ and *ε*″, the Cole–Cole curves of the N-doped SiC nanowires were plotted, as shown in Figure 4. which illustrate the loss mechanism of the EMW. It can be clearly seen from the figure that a distinct tail appears in both types of samples, except for the semicircle representing the polarization relaxation loss. It implies that a considerable conductivity loss was produced in both N-doped SiC nanowires. This can be attributed to the fact that n-type doping increases the electrical conductivity of the SiC nanowires while forming a conductivity loss network. It is worth noting that the length of the tail of SN-C is larger than that of the SN-B sample, which also implies that the former has a higher conductivity loss performance.

**Figure 5:**
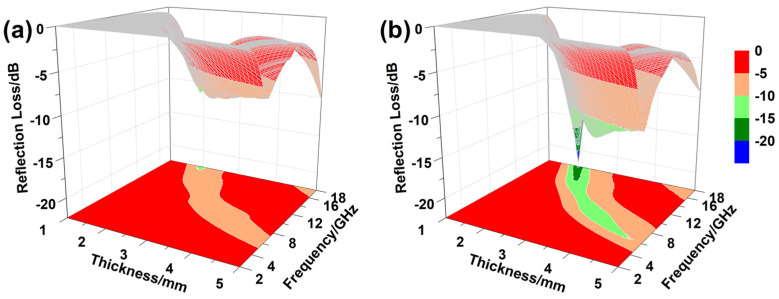
Three-dimensional patterns of reflection loss of N-doped SiC nanowires versus the absorber thickness and frequency: (**a**) SN-B and (**b**) SN-C.

The reflection loss (*RL*) directly reflects the electromagnetic wave absorption ability of the material, which is calculated according to the transmission line theory. A reflection loss of less than −10 dB means that 90% of the electromagnetic wave is absorbed.

(3) $$Z_{in}=\sqrt{\frac{\mu_{r}}{\varepsilon_{r}}}tanh\left(j\frac{2\pi fd}{c}\sqrt{\mu_{r}\varepsilon_{r}}\right)$$

(4) $$RL=20log\left|\frac{Z_{in}-1}{Z_{in}+1}\right|$$

where *Z_in_* is the input impedance, *ε_r_* and *μ_r_* are the complex permittivity and permeability, respectively, *f* is the EMW frequency, *d* is the absorber thickness, and *c* is the light velocity. For the same absorber thickness, a lower reflection loss indicates a higher EMW absorption performance. Three-dimensional plots of the reflection loss of N-doped SiC nanowires versus the absorber thickness (1–5 mm) and frequency (2–18 GHz) were illustrated in Figure 5. According to the results of our previous study, the minimum reflection loss of the pristine SiC nanowires at the same filling ratio is greater than −10 dB [34]. From the calculation results of reflection loss, it can be seen that the EMW absorption performances of the SiC nanowires prepared by different N-doping methods are improved, but there are differences between them. The SN-B sample shows a minimum reflection loss of −11.49 dB@17.92 GHz, and an effective absorption (RL ≤ −10 dB) bandwidth of 0.96 GHz (range of 17.04–18 GHz) at the absorber layer thickness of 2.0 mm. Compared with the previously synthesized pristine SiC nanowires synthesized previously, its EMW absorption performance is slightly improved. In contrast, the EMW absorption performance of the SiC nanowires prepared using ammonium chloride as a doped nitrogen source was significantly improved. The SN-C sample exhibits a minimum reflection loss of −22.02 dB@17.92 GHz, and an effective absorption (RL ≤ −10 dB) bandwidth of 4.24 GHz (range of 13.76–18 GHz) at the absorber layer thickness of 2.2 mm. As mentioned above, the SN-C sample has a higher density of SFs as well as a higher concentration of N doping. Thus, the polarization loss due to stacking faults and the conductivity loss due to n-type doping synergistically improve the EMW absorption performance of the SN-C sample [35].

**Figure 6:**
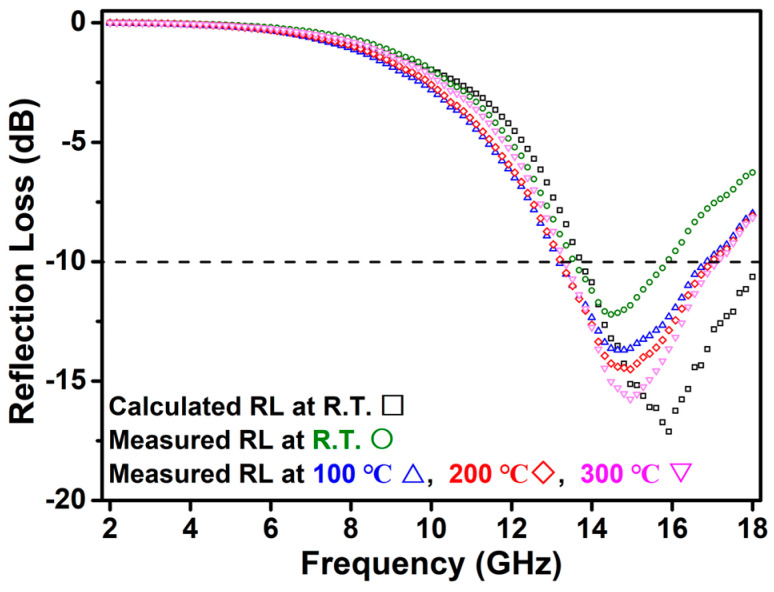
Reflection loss of SN-C SiC nanowires/resin composite coating (2.2 mm thickness) versus the frequency and temperature.

Based on the above analytical results, the measured reflection loss of the SN-C samples at high temperatures was further investigated. Figure 6 shows the measured reflection loss of the SN-C SiC nanowire/resin composite coating versus frequency and temperature. At room temperature, the measured EMW absorption performances are weaker than the calculation results. It is speculated that the particular morphology of the SiC nanowires makes them prone to agglomeration, resulting in them not being uniformly dispersed in the resin materials. On the one hand, the agglomeration of nanowires will lead to the reduced scattering of electromagnetic waves; on the other hand, the agglomeration of nanowires is not conducive to the formation of a conductive network, which will reduce the transport path of dissipative currents. Therefore, the actual EMW absorption performance of the SN-C sample was reduced. However, the EMW absorption performance of the composite coating is gradually enhanced with the increase in temperature. When the temperature is 300 °C, its minimum measured reflection loss reaches −15.86 dB@15.04 GHz and the effective absorption (RL ≤ −10 dB) bandwidth reaches 3.76 GHz (range of 13.32–17.08 GHz). This is attributed to the elevated temperature enhancing the conductivity loss of the SiC nanowires. The relationship between electrical conductivity and temperature can be described as the following equation [36]:

(5) $$\sigma \left(T\right)=Ae^{-E/2kT}$$

where A is a constant, *k* is the Boltzmann constant and *E* is the band gap between the conduction band and the impurity energy level. According to the Equations (1) and (4), the electrical conductivity *σ*(T) and conductivity loss will increase with increasing temperature, which in turn enhances the reflection loss of SiC nanowires. This result suggests that N-doped SiC nanowires are a potential high-temperature EMW absorbing material.

## 4. Conclusions

In summary, N-doped SiC nanowires were synthesized through three different doping methods. First, the synthesis of SiC nanowires by microwave heating under a nitrogen atmosphere will result in a mixture of Si_2_ON and SiC. Second, heat-treatment nitriding of SiC nanowires in a nitrogen atmosphere will significantly reduce the stacking fault density. Finally, the monophasic SiC nanowires with a higher stacking fault density and N-doping concentration were synthesized via microwave heating under an argon atmosphere by adding ammonium chloride as a nitrogen source to the raw materials. Therefore, the polarization loss induced by the stacking faults and the conductivity loss caused by doping synergistically enhance their dielectric properties and EMW absorption performance in the range of 2–18 GHz. When the filling ratio of N-doped SiC nanowires is 20 wt.%, the composite shows a minimum reflection loss of –22.2 dB@17.92 GHz, and an effective absorption (RL ≤ –10 dB) bandwidth of 4.24 GHz at the absorber layer thickness of 2.2 mm. It is worth noting that the EMW absorption properties of N-doped SiC nanowires are gradually enhanced with increasing temperature, indicating that it is an excellent high-temperature EMW absorbing material.

## Figures and Tables

**Table 1:** Preparation parameters of N-doped SiC nanowires.

Sample	Microwave Synthesis			Second Heat Treatment	
	Temperature × Time	Addition	Atmosphere	Temperature × Time	Atmosphere
SN-A	1500 °C × 30 min	-	N_2_	-	-
SN-B	1500 °C × 30 min	-	Ar	1200 °C × 30 min	N_2_
SN-C	1500 °C × 30 min	NH_4_Cl	Ar	-	-

**Table 2:** Lattice parameters and SF density of the N-doped SiC nanowires.

Sample	*A*-Lattice (Å)	Unit Cell Volume (Å^3^)	SF Density
Pristine SiC nanowires [34]	4.356	82.65	2.70
SN-B	4.350	82.31	2.06
SN-C	4.352	82.42	2.34

## Data Availability

Not applicable.
